# Dissociable cognitive impairments in two strains of transgenic Alzheimer’s disease mice revealed by a battery of object-based tests

**DOI:** 10.1038/s41598-018-37312-0

**Published:** 2019-01-11

**Authors:** Samantha D. Creighton, Ari L. Mendell, Daniel Palmer, Bettina E. Kalisch, Neil J. MacLusky, Vania F. Prado, Marco A. M. Prado, Boyer D. Winters

**Affiliations:** 1 Department of Psychology and Collaborative Neuroscience Program, University of Guelph, Guelph, ON Canada; 2 Department of Biomedical Sciences and Collaborative Neuroscience Program, University of Guelph, Guelph, ON Canada; 3 Molecular Medicine Research Group, Robarts Research Institute, Schulich School of Medicine & Dentistry, University of Western Ontario, London, Ontario, Canada; 4 Department of Physiology & Pharmacology, Schulich School of Medicine & Dentistry, University of Western Ontario, London, Ontario, Canada; 5 Department of Anatomy & Cell Biology, Schulich School of Medicine & Dentistry, University of Western Ontario, London, Ontario, Canada

## Abstract

Object recognition tasks detect cognitive deficits in transgenic Alzheimer’s disease (AD) mouse models. Object recognition, however, is not a unitary process, and there are many uncharacterized facets of object processing with relevance to AD. We therefore systematically evaluated object processing in 5xFAD and 3xTG AD mice to clarify the nature of object recognition-related deficits. Twelve-month-old male and female 5xFAD and 3xTG mice were assessed on tasks for object identity recognition, spatial recognition, and multisensory object perception. Memory and multisensory perceptual impairments were observed, with interesting dissociations between transgenic AD strains and sex that paralleled neuropathological changes. Overreliance on the widespread “object recognition” task threatens to slow discovery of potentially significant and clinically relevant behavioural effects related to this multifaceted cognitive function. The current results support the use of carefully designed object-based test batteries to clarify the relationship between “object recognition” impairments and specific aspects of AD pathology in rodent models.

## Introduction

Alzheimer’s disease (AD) is a neurodegenerative disorder characterized by cognitive dysfunction, as well as pathological accumulation of amyloid-β (Aβ) and hyperphosphorylated tau^1^. Transgenic mouse models of AD that express familial AD mutations recapitulate key features of Aβ and tau pathology and provide valuable insight into molecular and behavioural abnormalities in AD patients. 5xFAD transgenic mice express three mutations in the APP gene (K670N/M671L, I716V, and V717I) and two mutations in the PS1 gene (M146L and L286V), which induces aggressive amyloid pathology and neuronal loss^2^. 3xTG transgenic mice express APP (K670N/M671L) and PS1 (M146V) mutations associated with amyloid pathology in addition to a human tauopathy mutation (tauP301L)^3^. Because 5xFAD and 3xTG models have distinct neuropathological features and staging, comparing these complementary AD models could help clarify behavioural deficits associated with specific elements of AD pathology.

Object recognition (OR) is a rodent analogue of visual recognition memory tests in which human AD patients are impaired^2,3^. Rodent OR tasks have great translational potential because extensive training, reward and exposure to aversive stimuli are not required, and their one-trial nature is analogous to human episodic memory. Several studies have demonstrated OR memory deficits in 5xFAD^4–9^ and 3xTG mice^10–24^. However, the specific nature of OR deficits remains somewhat unclear, as some studies have failed to report OR impairments^8,13,17,25–29^. Differential performance on OR tasks is likely due to the lack of a standardized OR paradigm and systematic evaluation of sex, age and mnemonic delay. Furthermore, procedural differences can alter the conceptual nature of the task and thus tax distinct behavioural processes. Indeed, there are also many specific facets of object information processing that are affected by AD neuropathology in human patients. For example, AD patients have impaired spatial processing^30^, as well as multisensory integration and perception deficits^31,32^. While studies have described deficits in object spatial processing in transgenic AD mice^33^, multisensory integration has yet to be evaluated.

Here, we used a battery of specifically-designed cognitive tests to perform a systematic analysis of object processing in 12-month-old male and female 5xFAD and 3xTG mice to clarify the relationship between pathology and behavioural deficits across strains of AD mice. We report multifaceted impairment in object processing, with interesting strain and sex differences in object identity memory (OR), spatial memory (object location; OL), and multisensory perceptual integration (multisensory object oddity; MSO) that correspond with pathological changes in Aβ42, phosphorylated tau (P-tau) and other proteins associated with AD pathology.

## Results

### Object-based Memory Tasks

**Figure 1:**
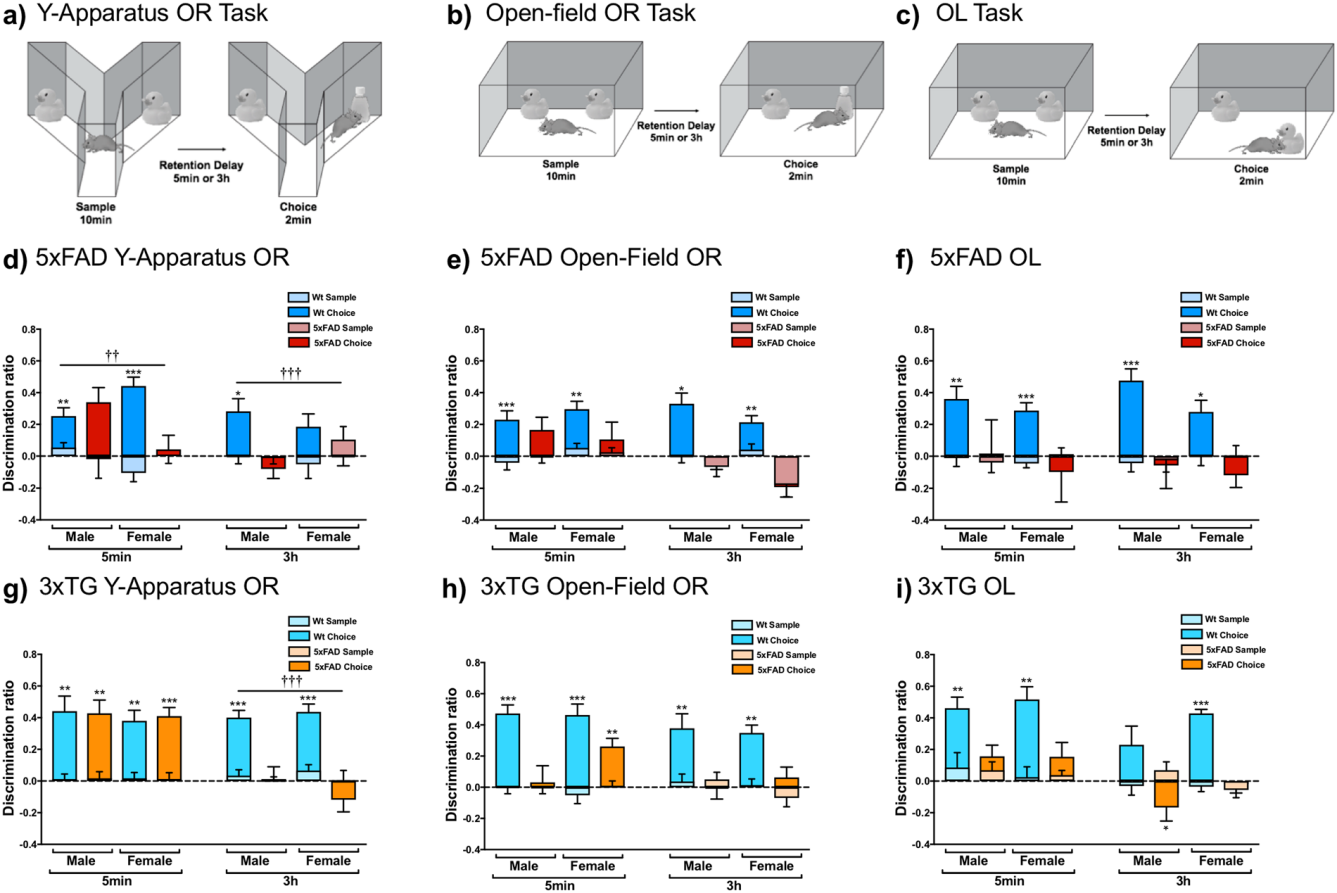
5xFAD and 3xTG mice have impairments in object-based memory tasks. Schematic representations of the (**a**) Y-apparatus OR, (**b**) open-field OR, and (**c**) OL tasks. (**d**) 5xFAD mice were impaired on Y-apparatus OR (Wt male n = 11, Wt female n = 8, 5xFAD male n = 10, 5xFAD female n = 11; significant main effects of genotype (F_1,36_ = 19.198, p < 0.001) and delay (F_1,36_ = 9.560, p = 0.004), as well as a genotype x delay interaction (F_1,36_ = 8.563; p = 0.006) were observed), (**e**) open-field OR (Wt male n = 11, Wt female n = 8, 5xFAD male n = 10, 5xFAD female n = 11; significant main effects of genotype (F_1,32_ = 6.392, p = 0.017) and delay (F_1,32_ = 22.725, p < 0.001), as well as a genotype x delay interaction (F_1,32_ = 7.791, p = 0.009) were found) and (**f**) OL (Wt male n = 15, Wt female n = 12, 5xFAD male n = 10, 5xFAD male n = 10; a significant main effect of genotype was found (F_1,43_ = 20.451, *p* < 0.001) at both 5 min and 3 h retention delays. 3xTG mice had more selective memory impairments. (**g**) 3xTG mice were impaired on Y-apparatus OR at the 3 h, but not 5 min, delay (Wt male n = 11, Wt female n = 12, 3xTG male n = 7, 3xTG female n = 10; significant main effects of genotype (F_1,36_ = 26.260, p < 0.001) and delay (F_1,36_ = 19.172, p < 0.001), as well as a genotype x delay interaction (F_1,36_ = 21.012, p < 0.001) were observed). (**h**) 3xTG males were impaired on open-field OR at both the 5 min and 3 h delays, whereas females were only impaired at the 3 h delay (Wt male n = 11, Wt female n = 12, 3xTG male n = 7, 3xTG female n = 12; significant main effects of genotype (F_1,36_ = 26.260, p < 0.001) and delay (F_1,36_ = 19.172, p < 0.001), as well as a genotype x delay interaction (F_1,36_ = 21.012, p < 0.001) were demonstrated). (**i**) 3xTG mice were also impaired on OL at both delays (Wt male n = 12, Wt female n = 15, 3xTG male n = 14, 3xTG female n = 15; significant main effects of genotype (F_1,51_ = 43.417, p < 0.001) and delay (F_1,51_ = 16.387, p < 0.001) were found).Data are mean discrimination ratio ± SEM. *p < 0.05, **p < 0.01, ***p < 0.001 indicates significant differences between sample and choice DR, suggesting intact memory (paired-sample t-tests, two-tailed). ^††^p < 0.01, ^†††^p < 0.001 indicates a significant overall difference between Wt and 5xFAD or 3xTG mice (independent samples t-test, two-tailed, Bonferroni correction).

To specifically evaluate object identity processing, 5xFAD and 3xTG mice were tested using the Y-apparatus, which minimizes contextual cues. Y-apparatus OR was impaired in a delay-independent manner in 5xFAD mice (2 × 2 × 2 split-plot ANOVA: genotype (F_1,36_ = 19.198, p < 0.001), delay (F_1,36_ = 9.560, p = 0.004), and genotype x delay (F_1,36_ = 8.563; p = 0.006); Fig. 1d). Paired sample t-tests between sample and choice DR suggest intact memory in Wt males and Wt females at 5 min (Wt males, 5 min: t_10_ = −3.253, p = 0.009; Wt males, 3 h: t_10_ = −2.787, p = 0.019; Wt females, 5 min: t_7_ = −6.195, p < 0.001), but impaired memory in Wt females at 3 h (although approaching statistical significance; t_7_ = 2.071, p = 0.054), and 5xFAD mice in all conditions, as they failed to discriminate between the novel and sample objects (5xFAD males, 5 min: t_9_ = −2.062, p = 0.069 (although approaching significance); 5xFAD males, 3 h: t_9_ = 1.009, p = 0.339; 5xFAD females, 5 min: t_10_ = −0.407, p = 0.692; 5xFAD females, 3 h: t_10_ = 0.870, p = 0.405). 3xTG mice, however, were impaired in a delay-dependent manner (2 × 2 × 2 split-plot ANOVA: genotype (F_1,36_ = 26.260, p < 0.001), delay (F_1,36_ = 19.172, p < 0.001), and genotype x delay (F_1,36_ = 21.012, p < 0.001); Fig. 1g). Paired samples t-tests indicated intact memory in Wt mice (Wt males, 5 min: t_10_ = −4.453, p = 0.001; Wt males, 3 h: t_10_ = −6.851, p < 0.001; Wt females, 5 min: t_11_ = −4.013, p = 0.002; Wt females, 3 h: t_11_ = −6.851, p < 0.001), and 3xTG mice at 5 min (3xTG males: t_6_ = −4.321, p = 0.005; 3xTG females: t_9_ = −8.264, p < 0.001), but impaired memory in 3xTG mice at 3 h, as they failed to distinguish significantly between novel and sample objects (3xTG males: t_6_ = −0.135, p = 0.897; 3xTG females: t_9_ = 0.963, p = 0.361).

We also evaluated OR in 5xFAD and 3xTG mice using a standard open-field, where spatial information is readily available. 5xFAD mice were also delay-independently impaired on open-field OR (2 × 2 × 2 split-plot ANOVA: genotype (F_1,32_ = 6.392, p = 0.017), delay (F_1,32_ = 22.725, p < 0.001), and genotype × delay (F_1,32_ = 7.791, p = 0.009); Fig. 1e). Paired samples t-tests between sample and choice DR indicated intact memory in Wt mice (Wt males, 5 min: t_10_ = −6.219, p < 0.001; Wt males, 3 h: t_10_ = −4.346, p = 0.002; Wt females, 5 min: t_7_ = −4.657, p = 0.002; Wt females, 3 h: t_7_ = −3.928, p = 0.006), but impaired memory in 5xFAD mice in all conditions, as they failed to distinguish significantly between novel and sample objects (5xFAD males, 5 min: t_9_ = −2.070, p = 0.068 (although approaching statistical significance); 5xFAD males, 3 h: t_10_ = t_9_ = −0.415, p = 0.689; 5xFAD females, 5 min: t_10_ = −0.668, p = 0.519; 5xFAD females, 3 h: t_10_ = 0.335, p = 0.746). Open-field OR was also impaired in 3xTG mice, as females and males were delay-dependently and delay-independently impaired, respectively (2 × 2 × 2 split-plot ANOVA: genotype (F_1,36_ = 26.260, p < 0.001), delay (F_1,36_ = 19.172, p < 0.001), and genotype × delay (F_1,36_ = 21.012, p < 0.001); Fig. 1h). Paired samples t-tests indicated intact memory in Wt mice (Wt males, 5 min: t_10_ = 3.918, p = 0.001; Wt males, 3 h: t_10_ = 3.000, p = 0.008; Wt females, 5 min: t_11_ = −7.094, p < 0.001; Wt females, 3 h: t_11_ = 4.835, p = 0.001) and 3xTG females at 5 min (3xTG females: t_9_ = −4.096, p = 0.003), but impaired memory in 3xTG males at both delays (5 min: t_6_ = −0.413, p = 0.694; 3 h: t_6_ = 0.708, p = 0.505), and 3xTG females at 3 h (t_9_ = −1.230, p = 0.250).

To evaluate spatial object processing directly, 5xFAD and 3xTG mice were tested on the OL task. 5xFAD mice had impaired spatial memory at both 5 min and 3 h delays (2 × 2 × 2 split-plot ANOVA: genotype (F_1,43_ = 20.451, *p* < 0.001); Fig. 1f). Paired samples t-tests between sample and choice DR indicated intact memory in Wt mice (Wt males, 5 min: t_14_ = −4.115, p = 0.001; Wt males, 3 h: t_14_ = −6.771, p < 0.0001; Wt females, 5 min: t_11_ = −4.013, p = 0.002; Wt females, 3 h: t_11_ = −2.663, p = 0.026), but impaired memory in 5xFAD mice in all conditions (5xFAD males, 5 min: t_9_ = −0.228, p = 0.825; 5xFAD males, 3 h: t_9_ = 0.200, p = 0.846; 5xFAD females, 5 min: t_9_ = 0.522, p = 0.613; 5xFAD females, 3 h: t_9_ = 0.200, p = 0.846). Similarly, OL was delay-independently impaired in 3xTG mice (2 × 2 × 2 split-plot ANOVA: genotype (F_1,51_ = 43.417, p < 0.001), and delay (F_1,51_ = 16.387, p < 0.001); Fig. 1i). Paired samples t-tests suggest intact memory in Wt females and Wt males at 5 min (Wt males, 5 min: t_11_ = −3.850, p = 0.002; Wt females, 5 min: t_14_ = −5.264, p < 0.001; Wt females, 3 h; t_14_ = 10.856, p < 0.001), but impaired memory in Wt males at 3 h and 3xTG mice in all conditions (Wt males, 3 h: t_11_ = 1.978, p = 0.076 (although approaching significance); 3xTG males, 5 min: t_13_ = −1.251, p = 0.233; 3xTG females, 5 min: t_14_ = −1.149, p = 0.271; 3xTG females, 3 h: t_14_ = −0.576, p = 0.575). 3xTG males displayed a familiarity preference at 3 h (t_13_ = 2.518, p = 0.027), instead of the novelty preference from which rodent object memory is typically inferred. A familiarity preference may imply intact memory, and could reflect abnormities in habituation, dishabituation or novelty preference. However, given the impairment observed at the 5-min delay, in this case it is possible that the familiarity preference is the result of a sample DR greater than zero. Indeed, a single-sample t-test comparing the 3xTG male choice DR at 3 h to 0 was not significant (t_13_ = −2.103, p = 0.06).

### Perceptual Object Oddity Tasks

**Figure 2:**
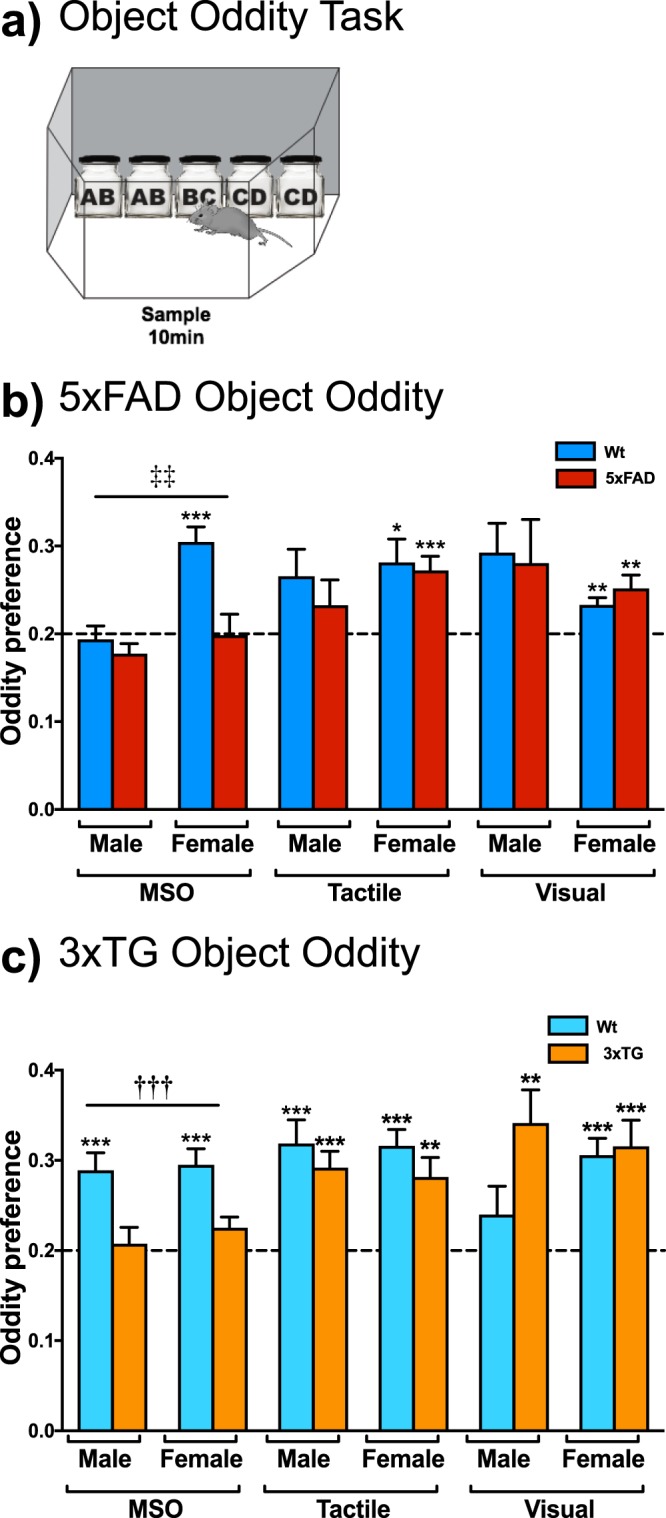
5xFAD and 3xTG mice have impairments in multisensory object oddity. (**a**) Schematic of multisensory and unimodal object oddity tasks. Wt and 5xFAD male mice had irregular performance on multisensory and unimodal tactile and visual object oddity. (**b**) 5xFAD female mice had a deficit in multisensory but not unimodal tactile or visual oddity (Wt male n = 10, Wt female n = 10, 5xFAD male n = 12, 5xFAD female n = 11; a significant main effect of task (F_2,78_ = 3.740, p = 0.035) and a task x sex interaction (F_2,78_ = 4.302, p = 0.022) were observed). (**c**) 3xTG mice had deficits in multisensory but not unimodal tactile or visual object oddity (Wt male n = 13, Wt female n = 15, 3xTG male n = 14, 3xTG female n = 14; a significant main effect of task (F_2,104_ = 4.795, p = 0.010) and task x genotype interaction (F_1,104_ = 6.967, p = 0.001) were found). Data are mean oddity preference ± SEM. *p < 0.05, **p < 0.01, ***p < 0.001 indicates significant one-sample t-tests, suggesting intact perception (two-tailed). ^†††^p < 0.001 Indicates a significant difference between Wt and 3xTG mice (independent samples t-test, two-tailed, Bonferroni correction). ^‡‡^p < 0.01 Indicates a significant difference between males and females (independent samples t-test, two-tailed, Bonferroni correction).

Finally, object oddity tasks were used to evaluate multisensory integration, as well as unimodal tactile and visual object perception. Wt males and 5xFAD mice had irregular performance on object oddity (2 × 2 × 3 split-plot ANOVA: task (F_2,78_ = 3.740, p = 0.035) and task × sex (F_2,78_ = 4.302, p = 0.022); Fig. 2b). One-sample t-tests suggested intact perception in Wt females and Wt males (Wt male, visual: t_9_ = 2.749, p = 0.023; Wt female, MSO: t_9_ = 6.018, p < 0.001; Wt female, visual: t_9_ = 4.4092, p = 0.003; Wt female, tactile: t_9_ = 3.062, p = 0.014), with the exception of Wt males on the MSO and tactile tasks (Wt male, MSO: t_9_ = −0.409, p = 0.692; Wt male, tactile: t_9_ = 2.123, p = 0.066 (although approaching statistical significance)). 5xFAD female mice had intact unimodal perception (visual: t_10_ = 3.366, p = 0.007; tactile: t_10_ = 4.502, p = 0.001), but not MSO (t_10_ = −0.058, p = 0.955). 5xFAD males had impaired perception on all tasks (MSO: t_11_ = −1.978, p = 0.073 (although approaching significance); visual: t_11_ = 1.619, p = 0.134; tactile: t_11_ = 1.127, p = 0.286). 3xTG mice demonstrated a selective impairment in tactile-visual multisensory perception, while unimodal tasks were generally unimpaired (2 × 2 × 3 split-plot ANOVA: task (F_2,104_ = 4.795, p = 0.010) and task x genotype (F_1,104_ = 6.967, p = 0.001); Fig. 2c). One-sample t-tests demonstrated perception above chance in Wt mice (males, MSO: t_12_ = 4.572, p = 0.001; males, tactile; t_12_ = 4.535, p = 0.001; females, MSO: t_14_ = 5.333, p < 0.001; females, visual: t_14_ = 5.627, p < 0.001; females, tactile: t_14_ = 6.460, p < 0.001), with the exception of visual perception in Wt males (visual: t_12_ = 1.274, p = 0.220). 3xTG mice had intact perception (3xTG males, visual: t_13_ = 3.609, p = 0.003; 3xTG males, tactile: t_13_ = 5.065, p < 0.001; 3xTG females, visual: t_13_ = 4.027, p = 0.001; 3xTG females, tactile t_13_ = 3.788, p = 0.002), except on the MSO task (males: t_13_ = 0.401, p = 0.695; females: t_13_ = 2.156, p = 0.050 (although very close to significance)).

### ELISA

**Figure 3:**
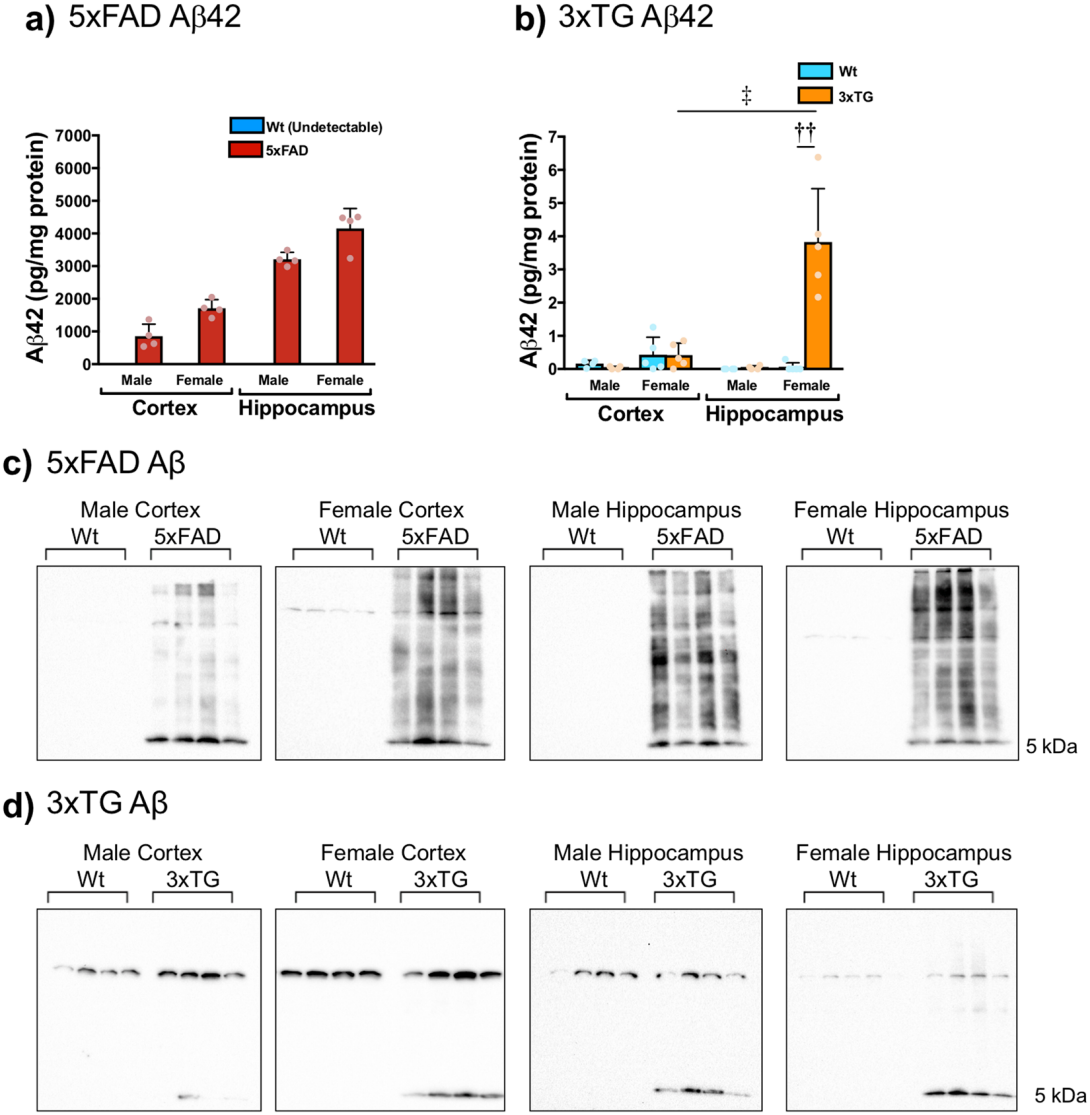
Aβ42 deposition in 13 month old 5xFAD and 3xTG mice. (**a**) There is extensive Aβ42 accumulation in the cortex and HPC of 5xFAD mice (n = 4 per group; main effects of genotype (F_1,24_ = 632.278, p < 0.0001), sex (F_1,24_ = 20.770, p < 0.0001), brain region (F_1,24_ = 147.648, p < 0.0001), as well as genotype x brain region (F_1,24_ = 147.648, p < 0.0001), and genotype × sex (F_1,24_ = 20.770, p < 0.0001) interactions were observed). (**b**) Only 3xTG female mice demonstrate sizeable Aβ42 deposition in the HPC (male n = 4 per group, female n = 5 per group; significant main effects of genotype (F_1,28_ = 17.329, p < 0.0001), sex (F_1,28_ = 25.974, p < 0.0001), brain region (F_1,28_ = 10.650, p = 0.003), as well as genotype x sex (F_1,28_ = 20.093, p < 0.0001), genotype x brain region (F_1,28_ = 20.093, p < 0.0001), sex x brain region (F_1,28_ = 20.093, p < 0.001), and genotype x sex x brain region (F_1,28_ = 16.816, p < 0.0001) interactions were observed). Total Aβ (37, 38, 39, 40, 42), however, could be detected in both (**c**) 5xFAD and (**d**) 3xTG mice, above Wt levels, using western blotting. ELISA data are mean Aβ42 pg/ug protein ± SEM. ^†††^p < 0.001 Indicates a significant difference between Wt and 5xFAD or 3xTG mice (independent samples t-test, two-tailed, Bonferroni correction).

5xFAD mice have extensive Aβ42 deposition in the cortex and HPC (2 × 2 × 2 ANOVA: genotype (F_1,24_ = 632.278, p < 0.0001), sex (F_1,24_ = 20.770, p < 0.0001), brain region (F_1,24_ = 147.648, p < 0.0001), genotype x brain region (F_1,24_ = 147.648, p < 0.0001), and genotype × sex (F_1,24_ = 20.770, p < 0.0001); Fig. 3a). Note: Aβ42 was undetectable in all Wt samples (<10 pg/mL); values of 0 were assigned to undetectable samples for analyses. Only 3xTG female mice demonstrate sizeable Aβ42 deposition in the HPC (2 × 2 × 2 ANOVA: genotype (F_1,28_ = 17.329, p < 0.0001), sex (F_1,28_ = 25.974, p < 0.0001), brain region (F_1,28_ = 10.650, p = 0.003), genotype × sex (F_1,28_ = 20.093, p < 0.0001), genotype x brain region (F_1,28_ = 20.093, p < 0.0001), sex × brain region (F_1,28_ = 20.093, p < 0.001), and genotype x sex x brain region (F_1,28_ = 16.816, p < 0.0001); Fig. 3b). Independent samples t-tests revealed significantly higher Aβ42 levels in the 3xTG female HPC than the Wt HPC (t_8_ = −5.222, p < 0.0001) and 3xTG female cortex (t_8_ = 4.624, p = 0.001). Note: Aβ42 was undetectable (<1 pg/mL) in 4 Wt male HPC samples, 4 Wt female HPC samples, 2 3xTG male cortical samples, 1 3xTG female cortical sample, and two 3xTG male HPC samples. Although significant Aβ42 was undetectable by ELISA in 3xTG males, total Aβ (37,38,39,40,42) could be detected in both 3xTG females and males, above Wt levels, using western blotting (Fig. 3d). Densitometry was not performed because Aβ was undetectable in Wt samples.

### Western Blots

Aged 5xFAD and 3xTG mice have several significant changes to a number of proteins associated with AD pathology.

**Figure 4:**
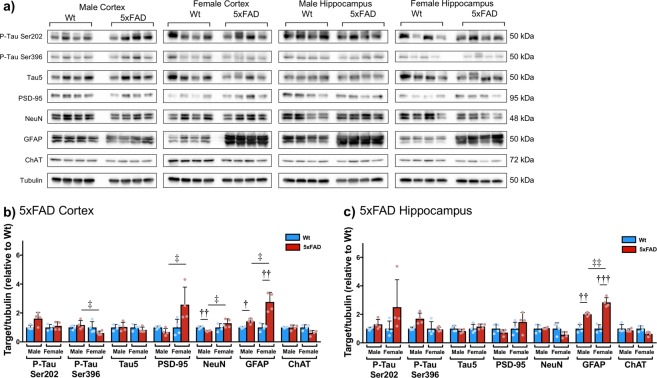
13 month old 5xFAD mice have abnormal expression of phospho-tau (Ser202, Ser396), PSD-95, NeuN, GFAP, and ChAT in the cortex and/or HPC. (**a**) Representative western blots (n = 4 per group). The approximate molecular weights for each identified protein, in kilodaltons (kDa) are shown to the right of the bands. Corresponding densitometric measurement in the (**b**) cortex (P-tau Ser202 genotype (F_1,16_ = 5.633, p = 0.035); P-tau Ser396 sex (F_1,16_ = 5.635, p = 0.035), genotype × sex (F_1,16_ = 4.770, p = 0.050); PSD-95 sex (F_1,16_ = 9.742, p = 0.009); NeuN sex (F_1,16_ = 6.702, p = 0.024), genotype x sex (F_1,16_ = 7.344, p = 0.019); GFAP genotype (F_1,16_ = 48.595, p < 0.0001), sex (F_1,16_ = 13.261, p = 0.003), genotype x sex (F_1,16_ = 13.995, p = 0.003); ChAT genotype (F_1,16_ = 6.432, p = 0.026), sex (F_1,16_ = 5.954, p = 0.031), genotype × sex (F_1,16_ = 5.612, p = 0.035) and (**c**) HPC (P-tau Ser202 genotype F_1,16_ = 143.914, p < 0.0001; GFAP genotype (F_1,16_ = 100.387, p < 0.0001), sex (F_1,16_ = 5.095, p = 0.043), genotype x sex (F_1,16_ = 5.090, p = 0.044); ChAT genotype (F_1,16_ = 7.109, p = 0.021). All values for 5xFAD mice are presented as a fold change in band intensity relative to Wt mice on the same blot; therefore, all Wt values are presented with a mean of 1. Data are mean expression ± SEM. ^††^p < 0.01 Indicates a significant difference between Wt and 5xFAD (independent samples t-test, two-tailed, Bonferroni correction). ^‡^p < 0.05 Indicates a significant difference between males and females (independent samples t-test, two-tailed, Bonferroni correction). Blots have been cropped for conciseness; full length blots are presented in Supplementary Figures 1–4.

5xFAD mice have abnormal expression of P-tau Ser202, P-tau Ser396, PSD-95, NeuN, GFAP, and ChAT, relative to Wt, in the cortex (2 × 2 ANOVA: P-tau Ser202 genotype (F_1,16_ = 5.633, p = 0.035); P-tau Ser396 sex (F_1,16_ = 5.635, p = 0.035), genotype x sex (F_1,16_ = 4.770, p = 0.050); PSD-95 sex (F_1,16_ = 9.742, p = 0.009); NeuN sex (F_1,16_ = 6.702, p = 0.024), genotype x sex (F_1,16_ = 7.344, p = 0.019); GFAP genotype (F_1,16_ = 48.595, p < 0.0001), sex (F_1,16_ = 13.261, p = 0.003), genotype x sex (F_1,16_ = 13.995, p = 0.003); ChAT genotype (F_1,16_ = 6.432, p = 0.026), sex (F_1,16_ = 5.954, p = 0.031), genotype x sex (F_1,16_ = 5.612, p = 0.035) Fig. 4b) and/or HPC (2 × 2 ANOVA: P-tau Ser202 genotype F_1,16_ = 143.914, p < 0.0001; GFAP genotype (F_1,16_ = 100.387, p < 0.0001), sex (F_1,16_ = 5.095, p = 0.043), genotype x sex (F_1,16_ = 5.090, p = 0.044); ChAT genotype (F_1,16_ = 7.109, p = 0.021) Fig. 4c)). Independent samples t-tests revealed a significant difference between cortical levels of P-tau Ser396 (t_6_ = 3.685, p = 0.010), PSD-95 (t_6_ = −4.206, p = 0.006), NeuN (t_6_ = −3.810, p = 0.009), and GFAP (t_6_ = −4.460, p = 0.004). There was also a significant difference between hippocampal levels of P-tau Ser396 in 5xFAD males and females (t_6_ = −5.117, p = 0.002). Independent samples t-tests revealed a significant difference between cortical levels of NeuN (t_6_ = 5.278, p = 0.002) in Wt and 5xFAD males. There was also a significant difference between Wt and 5xFAD levels of GFAP in the cortex (males: t_6_ = −4.930, p = 0.003; females: t_6_ = −5.669, p = 0.001) and HPC (males: t_6_ = −5.855, p = 0.001; females: t_6_ = −8.198, p < 0.0001).

**Figure 5:**
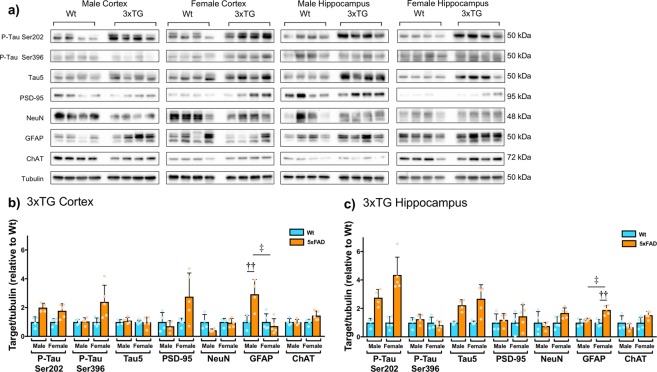
13 month old 3xTG mice have abnormal protein expression of phospho-tau (Ser202, Ser396 and total Tau5), PSD-95, NeuN, GFAP and ChAT in the cortex and/or HPC. (**a**) Representative western blots (male n = 4 per group, female n = 5 per group). Corresponding densitometric measurement in the (**b**) cortex (P-tau Ser202 genotype (F_1,18_ = 25.208, p < 0.0001); P-tau Ser396 genotype (F_1,18_ = 6.794, p = 0.021), sex (F_1,18_ = 5.855, p = 0.030), genotype x sex (F_1,18_ = 6.103, p = 0.027); PSD-95 sex (F_1,18_ = 5.876, p = 0.029), genotype x sex (F_1,18_ = 4.894, p = 0.044); NeuN genotype (F_1,18_ = 5.179, p = 0.039); GFAP sex (F_1,18_ = 10.885, p = 0.005), genotype x sex (F_1,18_ = 10.434, p = 0.006) and (**c**) HPC (P-tau Ser202 genotype (F_1,18_ = 77.628, p < 0.0001); tau5 genotype (F_1,18_ = 35.523, p < 0.0001); GFAP genotype (F_1,18_ = 23.200, p < 0.0001), sex (F_1,18_ = 7.261, p = 0.017), genotype × sex (F_1,18_ = 7.210, p = 0.018); ChAT sex (F_1,18_ = 6.468, p = 0.023), genotype × sex (F_1,18_ = 6.142, p = 0.027). All values for 3xTG mice are presented as a fold change in band intensity relative to Wt mice on the same blot; therefore, all Wt values are presented with a mean of 1. Data are mean expression ± SEM. ^††^p < 0.01 Indicates a significant difference between Wt and 3xTG (independent samples t-test, two-tailed, Bonferroni correction). ^‡^p < 0.05 Indicates a significant difference between males and females (independent samples t-test, two-tailed, Bonferroni correction). Blots have been cropped for conciseness; full length blots are presented in Supplementary Figures 5–8.

3xTG mice have abnormal protein expression of phospho-tau (Ser202, Ser396 and total Tau5), PSD-95, NeuN, GFAP and ChAT in the cortex (2 × 2 ANOVA: P-tau Ser202 genotype (F_1,18_ = 25.208, p < 0.0001); P-tau Ser396 genotype (F_1,18_ = 6.794, p = 0.021), sex (F_1,18_ = 5.855, p = 0.030), genotype x sex (F_1,18_ = 6.103, p = 0.027); PSD-95 sex (F_1,18_ = 5.876, p = 0.029), genotype x sex (F_1,18_ = 4.894, p = 0.044); NeuN genotype (F_1,18_ = 5.179, p = 0.039); GFAP sex (F_1,18_ = 10.885, p = 0.005), genotype x sex (F_1,18_ = 10.434, p = 0.006); Fig. 5b) and/or HPC (2 × 2 ANOVA: P-tau Ser202 genotype (F_1,18_ = 77.628, p < 0.0001); tau5 genotype (F_1,18_ = 35.523, p < 0.0001); GFAP genotype (F_1,18_ = 23.200, p < 0.0001), sex (F_1,18_ = 7.261, p = 0.017), genotype x sex (F_1,18_ = 7.210, p = 0.018); ChAT sex (F_1,18_ = 6.468, p = 0.023), genotype x sex (F_1,18_ = 6.142, p = 0.027); Fig. 5c). Independent samples t-tests revealed significant differences between cortical GFAP levels in Wt and 3xTG males (t_6_ = −3.561, p = 0.012), as well as male and female 3xTG mice (t_6_ = 4.394, p = 0.003). There was also a significant difference between hippocampal levels of GFAP in Wt and 3xTG females (t_6_ = −5.502, p = 0.001), as well as male and female 3xTG mice (t_6_ = −4.409, p = 0.003).

## Discussion

This study demonstrates impairments in object memory and perception in male and female 5xFAD and 3xTG mice, and these deficits parallel AD-like pathological changes. Specifically, tasks that directly probe different aspects of object processing (object identity memory, spatial location memory, and perceptual discrimination) reveal distinctive patterns of impairment in different strains and sexes. The pattern of impairments we observed in 5xFAD and 3xTG mice are consistent with both the large literature demonstrating impairments in AD mice^4–24^, as well as the lack of impairments^8,13,17,25–29^ seen in some studies which have used short retention delays or younger animals.

Object recognition impairments in 5xFAD and 3xTG mice were observed in both the Y-apparatus and open-field tasks. 5xFAD mice had OR impairments at the 5-min and 3-h retention delays, although a few 5xFAD groups appeared to be trending towards statistical limits that would demonstrate intact memory. The widespread impairments observed in 5xFAD mice are consistent with their severe pathology and possibly indicate the presence of attentional deficits in addition to memory impairment. 3xTG mice had selective impairments in Y-apparatus OR at 3 h, suggestive of a more selective long-term object memory deficit. However, when OR was evaluated in the open-field, 3xTG females were also selectively impaired at 3 h but 3xTG males were impaired at both 5 min and 3 h. This suggests that OR impairments can become more severe in some strains when the spatial nature of the task is increased. The Y-apparatus could increase the focus on objects and provides fewer potentially interfering contextual stimuli than the open-field. Perhaps AD mice have insufficient mnemonic resources for both object and environmental information present in the open-field OR task. While the HPC seems to be necessary for basic OR in mice^34,35^, the open-field OR task likely places even greater demand on the HPC^36,37^, which has substantial amyloid accumulation.

In OR, we also observed a subtle sex difference between 3xTG males and females, where 3xTG females had a less severe OR deficit than males. Specifically, short-term OR memory was intact in 3xTG females in open-field and Y-apparatus OR, whereas 3xTG males had short-term open-field OR deficits that were not observed at short retention delays in the Y-apparatus. This pattern is consistent with sexual dimorphism in working memory, such that 3xTG males have a more severe behavioural deficit beginning at two-months-of-age^38^. Although our data, and others^39–41^, demonstrate a more aggressive amyloid pathology in 3xTG females, activational estrogens are believed to be neuroprotective against Alzheimer’s pathology. Ovariectomy-induced depletion of sex hormones in 3xTG females increases Aβ accumulation and impairs HPC-dependent memory, an effect that can be attenuated by estrogen treatment^42–45^. It is also possible that differences in male and female longevity contributed to sex differences in 3xTG mice, since 3xTG females have a longer lifespan than males^46^. This sex difference was not observed in 5xFAD mice at this age, where a more aggressive amyloid pathology may block the beneficial effects of estrogens in easier testing conditions.

Object location memory was impaired across sex and AD strain, suggesting that spatial processing of objects may be more difficult for AD mice than the processing of object identity. Impaired OL memory is consistent with previous work^17,33,47–49^, and as OL is a HPC-dependent task this finding provides additional support for susceptibility of HPC-dependent processes to AD pathology. Since Wt males for the 3xTG strain had difficulty performing the OL task, it is possible that some OL deficits in males may be related to general aging rather than AD. Indeed, OL is often impaired in aged mice^50,51^.

Multisensory integration (MSI) is a relatively neglected facet of cognition in AD, despite findings from patients demonstrating abnormal MSI^31,32^. Multisensory integration involves the binding of information across sensory modalities, which can require connectivity of modality-specific sensory processing areas^52,53^. Given the disruption of cortical connectivity in human patients^54,55^ and APP/PS1 mice^56^, impaired connectivity between cortical regions may impair binding of visual and tactile object information. The current results represent the first demonstration of impaired MSI in rodent models of AD. While 5xFAD female and 3xTG mice show a selective deficit on tactile-visual MSO, 5xFAD and Wt male mice from the same background also had impairments in unimodal tactile and visual object oddity tasks. Unimodal tactile and visual object oddity performance also informs the interpretation of results from object memory tasks, in which mice must also encode object features perceptually. 5xFAD mice display delay-independent impairments on all mnemonic object tasks and 5xFAD males have impairments on tactile and visual oddity; therefore, impaired OR and OL in 5xFAD males could be most parsimoniously attributed to deficits in object perception rather than memory. Again, the impairment in 5xFAD Wt males may be related to accelerated aging or other abnormalities in the 5xFAD background strain. Conversely, 5xFAD females and 3xTG mice had selective multisensory oddity impairments, with spared unimodal perception; thus, it is unlikely that OR and OL impairments can be explained by perceptual deficits. In this case, the object test battery approach provides a great advantage over standard testing methods in this field by strengthening the conclusions that can be drawn about specific cognitive impairments.

Atypical motor and anxiety-like behaviours have been reported in both 5xFAD and 3xTG strains. Aged 3xTG mice have increased anxiety-like behaviours including increased restlessness, startle responses, and freezing^23,57–59^, whereas motor behaviour is more complex with accounts of hypoactivity^59^ or normal locomotion in the open-field^23^, as well as enhanced performance on other motor tasks^16,24^. Conversely, 5xFAD mice have reduced anxiety-like behaviours and motor deficits^60,61^. However, there were no consistent differences between genotypes or sex in terms of general object exploratory behaviour in any of the tests used; thus, it is unlikely that gross changes to motor or anxiety-related behaviors significantly impacted the current findings.

Abnormalities in several proteins associated with AD were seen in both 5xFAD and 3xTG mice. A pattern of amyloid deposition that is more severe in the HPC than cortex was observed in both mouse strains. 5xFAD mice have much more Aβ42 deposition than 3xTG mice, consistent with multiple APP and PS1 mutations acting in an additive fashion to accelerate amyloid deposition and cognitive impairment in the former^62^. While Aβ42 likely contributes to the observed cognitive impairments, especially in HPC-dependent object tasks, Aβ42 deposition is minimal in 3xTG mice at this advanced age. Indeed, Aβ42 was not elevated in the cortex or HPC of 3xTG males. However, we did detect increased levels of total Aβ, suggesting that 3xTG males may have different proportions of Aβ 37, 38, 39, 40, and 42 at 12-months-of-age. Despite the lack of robust changes in Aβ42, we have demonstrated significant behavioural impairments in male 3xTG mice, highlighting the importance of other pathological changes on behavioural phenotypes. Both strains had abnormalities in tau phosphorylation and astrogliosis, other pathological hallmarks of AD associated with cognitive impairment^63,64^. Consistent with previous observations we report elevations in tau phosphorylation in both 5xFAD^65–67^ and 3xTG^42,68–71^ mice, with novel strain, sex, and regional differences on phosphorylation at specific tau residues. Extensive gliosis (GFAP) was also observed in both 5xFAD^62,72^ and 3xTG^68–70,73^ mice, also with interesting differences between strain, sex and brain region. Paradoxically, PSD-95 was elevated in 5xFAD and 3xTG females which may result from the loss of PSD-95 at apical dendrites but accumulation in cell bodies in advanced AD^74^. Contrary to the existing literature^75^, ChAT was also elevated in 3xTG females and may reflect a compensatory response as female sex hormones can modulate AD pathology and cholinergic neurotransmission^42,76^. The opposite pattern was observed for ChAT in 5xFAD mice^77^.

The current study illustrates the subtle but potentially clinically significant differences in behavioural characterization possible when using a testing battery to probe different aspects of object information processing, not just “object recognition” as defined by the most commonly chosen task. We recommend such a systematic approach moving forward to better evaluate the cognitive phenotypes of transgenic models, as well as potential therapeutic interventions, in this context.

## Methods

### Animals

5xFAD wild-type (B6SJLF1/J; male n = 20, female n = 33) and transgenic (B6SJLTg(APPSwF1Lon,PSEN1*M146L*L286V)6799Vas/Mmjax; male n = 23, female n = 33) mice and 3xTG wild-type (B6129SF2/J; male n = 24, female n = 35) and transgenic (B6;129Psen1^tm1Mpm^ Tg(APPSwe, tauP301L)1Lfa/Mmjax; male n = 21, female n = 35) mice were obtained from Jackson Laboratory (Bar Harbor, USA). Because we were primarily comparing WT and TG mice, there was no random assignment to groups. A minimum of 7 mice per group were used for behavioural experiments; our previous work indicates power of 0.80 can be achieved with a sample size of 6 mice (α = 0.05). Differences in sample sizes between sexes was a result of attrition^46^. Mice were housed in polyethylene cages (16 × 12 × 26 cm) with corncob bedding, crink-l’Nest and cotton nest squares and food (Tekland Global 16% Protein Rodent Maintenance Diet, Harlan Tekland, USA) and water available *ad libitum*. Mice were tested during the light phase of a 12 h light/dark cycle (0800 lights on; 2000h lights off). All procedures followed the guidelines of the Canadian Council on Animal Care and were approved by the Animal Care Committee at the University of Guelph and the University of Western Ontario (2006-103 and 2006-104). Object recognition testing started when mice were 12 months of age after they were tested in different touchscreen tasks.

### Object-based Memory Tasks

Object recognition was run in a modified Y-apparatus with walls 30.5 cm high, and arms 15 cm long and 7 cm wide constructed from white Plexiglas (Fig. 1a). The start arm of the Y-apparatus has a guillotine door 11 cm from the back of the arm that was closed at the beginning of each trial. When OR is conducted in the Y-apparatus, spatial information is minimized, allowing for direct evaluation of object identity processing^37^. Objects were visually and tactually distinct, approximately 5–15 cm in height and 3–6 cm wide, made of glass, metal or plastic and were previously found to produce no obvious preference biases in mice. In the sample phase, mice were presented two identical objects to explore for 10 min. Following the sample phase, there was a 5-min or 3-h retention delay, to assess short- and long-term memory, respectively. At the end of the retention delay, mice underwent a 2-min choice phase, in which mice were presented with one object from the sample phase and a novel object. Object recognition was also evaluated with the same parameters in a square open-field arena (45 × 45 × 30 cm) made of white corrugated plastic (Fig. 1b). When OR is evaluated in the open-field, spatial and contextual cues in the testing room are readily available; this task might therefore be more hippocampus-dependent than Y-apparatus OR^37^. In both OR tasks, the order of object pairs, the designated sample and choice novel object within each pair, and the side of the apparatus (left or right) where the novel object was placed during the choice phase were counterbalanced. The object location task was used to evaluate spatial object memory in the open-field using the same testing parameters as OR, except that one object from the sample phase was moved to an adjacent corner of the arena in the choice phase (Fig. 1c); in this case, the location of one object, rather than its identity, is novel. In OL, the order of object pairs and the side of the apparatus (left or right) where the novel object was placed during the choice phase were counterbalanced. For all object memory tasks, the novelty preference was quantified by calculating a discrimination ratio (DR = (novel object exploration – familiar object exploration)/(total object exploration)). Repeated measures ANOVAs were used to analyze DRs with retention delay as a within-subjects factor and sex and genotype as between-subjects factors. Where appropriate, *post-hoc* t-tests were used to analyze group differences. In the sample phase, objects should be equally novel and a DR of approximately zero is expected. In the choice phase, a DR significantly greater than zero indicates novelty preference, from which we infer intact memory. Paired samples t-tests were used to compare sample and choice DRs, as a significant increase in the DR from sample to choice is indicative of intact memory. Outliers (>2 SD ± mean; 33 data points total) and mice that spent less than 3% of the choice phase exploring^78^ (19 data points) were excluded. General exploratory measures are reported in Supplementary Tables 3 and 4.

### Perceptual Object Oddity Tasks

Oddity tasks were run in a modified trapezoid-like open-arena (front wall 39 cm, side walls 14 cm, angled side walls 10 cm, back wall 28 cm; Fig. 2a). In MSO, mice explored two pairs of objects that shared combinations of tactile and visual features (e.g., AB/AB and CD/CD), as well as a dissimilar object that comprised a unique (i.e. ‘odd’) combination of those features (e.g., BC) for 10 min. Tactile object features were manipulated using varieties of sandpaper with different grades, while visual object features were manipulated using 2-D stickers with distinctive visual markings. Unimodal control trials were also run according to the same format, except the objects were distinguished by configurations of features from within the same sensory modality (i.e., visual or tactile)^52^. Placement of the ‘odd’ object was counterbalanced across trials and experiments.

An oddity preference for the ‘odd’ object was calculated (OP = (exploration of the odd object)/(total object exploration)). Repeated measures ANOVAs were used to analyze OPs with task as a within-subjects factor and genotype as a between-subjects factor. Where appropriate, *post-hoc* t-tests were used to analyze between group differences. With five objects, an OP significantly greater than 0.2 (‘chance’) indicates an oddity preference, from which we infer intact perceptual discrimination; this analysis was performed using one sample t-tests. Outliers (>2 SD ± mean) and mice that spent less than 3% of the sample phase exploring were excluded.

### General Behavioural Procedure

All behavioural testing was conducted under white fluorescent light in a 190 cm by 145 cm room with white walls and an orange door. A television, computer modem, and computer monitor sat atop metal shelving. A few additional visual cues were fixed to the walls. The apparatuses were placed on the floor away from the walls.

Prior to behavioural testing all mice were extensively handled and habituated to an empty testing apparatus for 10 min on two consecutive days. Behavioural testing began at least 24 h after the second habituation day. Immediately prior to testing, mice were brought into the testing room in their home cage. Mice were placed in either the start arm of the Y-apparatus or in a start box placed in the center of the open field. The trial began when the start arm/box was opened or removed, respectively, and the mouse began to explore. During testing, an experimenter blind to the experimental condition, viewed the mouse on a television screen and pressed a key corresponding to a given object at the onset and end of an exploratory bout (sniffing within 1 cm of the object and/or touching the object with the nose). Between trials, objects were wiped with 50% ethanol (to eliminate olfactory cues), the testing apparatus was wiped with dry paper towel and the mouse was returned to its home cage.

### Western Blots and ELISA

Immediately after behavioural testing (~13 months of age), cortices and hippocampi were homogenized in Triton X-100 lysis buffer containing 1 mM 4-(2-aminoethyl)benzenesulfonyl fluoride hydrochloride, 10 μM leupeptin, 25 μM aprotinin, 10 μM pepstatin A, and 700 units DNase. Lysate protein concentrations were determined using a Bradford assay.

Total Aβ (37, 38, 39, 40, 42), phospho-tau, total tau, NeuN, postsynaptic density (PSD)-95, glial fibrillary acidic protein (GFAP), and choline acetyltransferase (ChAT) levels were evaluated using Western blot analysis. For western blots protein samples (25 μg/well) were run on 10% SDS-PAGE using a Mini-PROTEAN Tetra cell system. Samples were transferred to nitrocellulose membranes, blocked in 3% BSA (Fisher Scientific; with or without milk) in tris-buffered saline 0.1% Tween20 (TBS-T) for 1.5 h, and incubated in primary antibodies overnight (see Supplementary Table 1). Blots were subsequently rinsed with TBS-T, incubated in secondary antibody in BSA, rinsed with TBS-T, and visualized using Luminata Forte chemi solution and the ChemiDoc MP imaging system. Densitometry was performed using Image Labv4.1 software comparing levels of each target to α-tubulin. To account for variability between blots the densitometric value for each individual protein band was expressed as a fraction of the total amount of that protein on the same blot. Each protein of interest was normalized to α-tubulin values from the same sample and the normalized protein levels in the transgenic mice were presented as the fold change relative to values from the Wt mice from the same blot. Two-way ANOVAs were used to analyze genotype and sex effects on each target protein. Log (x + 1) transformations were performed to address heterogeneity of variance.

ELISA measurements of Aβ42 were conducted using the standard or ultrasensitive amyloid beta 42 human ELISA kits from ThermoFisher Scientific for 5xFAD and 3xTG lysates, respectively. Three-way ANOVAs were used to analyze strain, genotype and sex effect on Aβ42.

### Statistical Analysis

All statistical analyses were performed with α = 0.05 using SPSS. Where appropriate, the Bonferroni correction was applied. All data met assumptions of normality and homogeneity.

## Supplementary information

Supplementary Material

## Data Availability

The datasets generated and analysed during the current study are available from the corresponding author on reasonable request.
