# Supplementary material for: Dissociable cognitive impairments in two strains of transgenic Alzheimer’s disease mice revealed by a battery of object-based tests
Source: Sci Rep. 2019 Jan 11;9:57. doi: 10.1038/s41598-018-37312-0 (PMC6329782; doi:10.1038/s41598-018-37312-0)
Supplement: Supplementary file 1 — Supplementary Material [file 41598_2018_37312_MOESM1_ESM.pdf]

# Dissociable cognitive impairments in two strains of transgenic Alzheimer's disease mice revealed by a battery of object-based tests

## Supplementary Material

**Samantha D. Creighton[a], Ari L. Mendell[b], Daniel Palmer[a,1,2], Bettina E. Kalisch[b], Neil J. MacLusky[b], Vania F. Prado[c,d,e], Marco A.M. Prado[c,d,e], Boyer D. Winters[a,*]**

[a]Department of Psychology and Collaborative Neuroscience Program, University of Guelph, Guelph, ON, Canada
[b]Department of Biomedical Sciences and Collaborative Neuroscience Program, University of Guelph, Guelph, ON, Canada
[c,1]Molecular Medicine Research Group, Robarts Research Institute, Schulich School of Medicine & Dentistry, University of Western Ontario, London, Ontario, Canada
[d,2]Department of Physiology & Pharmacology, Schulich School of Medicine & Dentistry, University of Western Ontario, London, Ontario, Canada
[e]Department of Anatomy & Cell Biology, Schulich School of Medicine & Dentistry, University of Western Ontario, London, Ontario, Canada

Supplementary Table 1
Western Blot Antibodies

| **Protein Target** | **Species Raised in; Monoclonal or Polyclonal** | **Dilution** | **Manufacturer and Catalog Number** |
|---|---|---|---|
| β-Amyloid (37,38,39,40,42) | Rabbit monoclonal | 1:1000 | Cell Signaling Technology, 8243 |
| Phospho-Tau Ser202 | Mouse polyclonal | 1:500 | Cell Signaling Technology, 11834 |
| Phospho-Tau Ser396 | Mouse monoclonal | 1:500 | Cell Signaling Technology, 9632 |
| Tau5 | Mouse polyclonal | 1:500 | Biolegend, 39413 |
| NeuN | Rabbit monoclonal | 1:1000 | Cell Signaling Technology, 24307 |
| Postsynaptic Density Protein 95 (PSD-95) | Mouse monoclonal | 1:400 | Santa Cruz Biotechnology, Inc., sc-32290 |
| Glial Fibrillary Acidic Protein (GFAP) | Mouse monoclonal | 1:10,000 | Cell Signaling Technology, 3670 |
| Choline acetyltransferase (ChAT) | Rabbit monoclonal | 1:1000 | Abcam, ab178850 |
| Goat anti-rabbit IgG- HRP secondary | Goat | 1:2500 | Santa Cruz Biotechnology Inc., sc-2004 |
| Goat nti-mouse IgG-HRP secondary | Goat | 1:2500 | Santa Cruz Biotechnology Inc., sc-2005 |

Supplementary Table 2
Total Object Exploration

| | | | | Total Mean Sample Exploration (s) | | Total Mean Choice Exploration (s) | |
|---|---|---|---|---|---|---|---|
| **Experiment** | | | | Wildtype | Transgenic | Wildtype | Transgenic |
| **Strain** | **Task** | **Sex** | **Delay** | | | | |
| **5xFAD** | **Y-apparatus OR** | **Male** | **5min** | 76.83 (10.45) | 88.61 (9.70) | 27.09 (5.99) | 17.10 (2.56) |
| | | | **3h** | 77.14 (11.62) | 72.25 (4.34) | 20.64 (2.47) | 18.90 (2.21) |
| | | **Female** | **5min** | 60.41 (4.17) | 67.96 (9.02) | 16.62 (1.92) | 20.21 (6.24) |
| | | | **3h** | 60.63 (4.52) | 73.48 (4.82) | 12.12 (1.43) | 21.83 (4.40) |
| | **Open-field OR** | **Male** | **5min** | 80.55 (5.66)* | 51.92 (7.60)* | 17.53 (1.27) | 12.35 (2.11) |
| | | | **3h** | 66.60 (4.16) | 46.44 (7.89) | 11.28 (1.39) | 10.09 (1.78) |
| | | **Female** | **5min** | 78.44 (6.01)* | 46.36 (5.35)* | 21.08 (2.00) | 15.27 (2.50) |
| | | | **3h** | 83.77 (15.36)* | 37.02 (7.87)* | 17.20 (1.94)** | 6.80 (1.20)** |
| | **OL** | **Male** | **5min** | 57.42 (2.43) | 64.54 (5.39) | 12.12 (0.99) | 10.85 (1.81) |
| | | | **3h** | 79.57 (3.22) | 71.61 (2.86) | 10.71 (1.59) | 10.94 (1.93) |
| | | **Female** | **5min** | 65.53 (4.59) | 51.74 (5.43) | 17.88 (2.07) | 14.44 (1.31) |
| | | | **3h** | 73.52 (7.13) | 55.09 (9.05) | 20.09 (1.85) | 10.88 (0.99) |
| | **MSO** | **Male** | | 29.94 (2.43) | 30.12 (2.91) | | |
| | | **Female** | | 36.03 (2.21) | 58.72 (6.33) | | |
| | **Tactile Oddity** | **Male** | | 44.35 (3.70) | 49.67 (4.72) | | |
| | | **Female** | | 47.24 (7.46)* | 84.26 (7.81)* | | |
| | **Visual Oddity** | **Male** | | 31.24 (5.30) | 31.67 (3.85) | | |
| | | **Female** | | 41.69 (2.60) | 58.21 (5.12) | | |
| **3xTG** | **Y-apparatus OR** | **Male** | **5min** | 53.26 (6.21) | 62.63 (3.03) | 22.10 (2.45) | 21.77 (2.23) |
| | | | **3h** | 55.28 (3.89) | 54.90 (4.70) | 22.99 (2.55) | 18.22 (2.15) |
| | | **Female** | **5min** | 55.12 (6.09) | 41.45 (4.66) | 20.23 (3.24) | 15.13 (1.86) |
| | | | **3h** | 47.24 (5.12) | 51.55 (6.18) | 18.01 (2.43) | 12.68 (1.73) |
| | **Open-field OR** | **Male** | **5min** | 53.81 (6.66) | 46.55 (5.25) | 14.40 (1.98) | 10.65 (1.46) |
| | | | **3h** | 48.98 (3.96) | 44.19 (5.05) | 9.11 (1.07) | 8.37 (1.50) |
| | | **Female** | **5min** | 49.28 (7.69) | 46.71 (9.03) | 13.52 (1.70) | 8.37 (1.05) |
| | | | **3h** | 52.37 (5.26) | 41.66 (5.69) | 12.32 (1.81) | 8.66 (1.05) |
| | **OL** | **Male** | **5min** | 32.77 (3.47) | 49.82 (5.00) | 9.60 (1.03) | 12.78 (1.09) |
| | | | **3h** | 47.33 (6.75) | 45.07 (3.02) | 7.79 (1.04) | 10.32 (1.74) |
| | | **Female** | **5min** | 44.32 (5.55) | 53.86 (3.97) | 17.74 (2.40) | 13.71 (1.14) |
| | | | **3h** | 59.50 (4.15) | 46.24 (4.35) | 14.93 (1.44) | 15.60 (1.58) |
| | **MSO** | **Male** | | 33.28 (4.10) | 36.88 (3.84) | | |
| | | **Female** | | 38.39 (2.59) | 37.76 (1.74) | | |
| | **Tactile Oddity** | **Male** | | 43.00 (4.91) | 45.95 (2.90) | | |
| | | **Female** | | 50.40 (4.83) | 42.11 (2.00) | | |
| | **Visual Oddity** | **Male** | | 30.38 (2.99) | 35.50 (2.89) | | |
| | | **Female** | | 34.22 (2.33) | 38.77 (1.97) | | |

Note. Paired samples t-tests between total sample exploration and novelty preference index (discrimination ratio or oddity preference) are not significant unless otherwise reported. Data are mean exploration (s) ± SEM, * $p < .05$, ** $p < .001$. There are few significant exploratory differences between Wt and transgenic mice.

Supplementary Table 3
Correlation Between Total Sample Object Exploration and OR and MSO Performance

| | | | | **Correlation Coefficient (*r*)** | |
|---|---|---|---|---|---|
| **Experiment** | | | | Wildtype | Transgenic |
| **Strain** | **Task** | **Sex** | **Delay** | | |
| **5xFAD** | **Y-apparatus OR** | **Male** | **5min** | .031 | .014 |
| | | | **3h** | .188 | -.195 |
| | | **Female** | **5min** | -.154 | .653 |
| | | | **3h** | .502 | -.037 |
| | **Open-field OR** | **Male** | **5min** | .320 | .298 |
| | | | **3h** | .042 | .024 |
| | | **Female** | **5min** | .169 | .400 |
| | | | **3h** | -.206 | .537 |
| | **OL** | **Male** | **5min** | .545 | -.052 |
| | | | **3h** | .469 | .195 |
| | | **Female** | **5min** | .164 | .143 |
| | | | **3h** | .334 | .047 |
| | **MSO** | **Male** | | -.286 | -.051 |
| | | **Female** | | -.418 | -.822 |
| | **Tactile Oddity** | **Male** | | .189 | .073 |
| | | **Female** | | .124 | -.100 |
| | **Visual Oddity** | **Male** | | .737 | -.154 |
| | | **Female** | | .169 | -.252 |
| **3xTG** | **Y-apparatus OR** | **Male** | **5min** | .106 | -.338 |
| | | | **3h** | .273 | -.113 |
| | | **Female** | **5min** | .379 | -.646 |
| | | | **3h** | -.006 | -.492 |
| | **Open-field OR** | **Male** | **5min** | -.385 | .587 |
| | | | **3h** | -.121 | -.367 |
| | | **Female** | **5min** | -.192 | -.398 |
| | | | **3h** | -.330 | .363 |
| | **OL** | **Male** | **5min** | .090 | .039 |
| | | | **3h** | -.135 | -.200 |
| | | **Female** | **5min** | .267 | -.167 |
| | | | **3h** | -.193 | -.291 |
| | **MSO** | **Male** | | .296 | .532 |
| | | **Female** | | -.178 | .347 |
| | **Tactile Oddity** | **Male** | | .091 | .469 |
| | | **Female** | | .566 | -.254 |
| | **Visual Oddity** | **Male** | | .500 | -.042 |
| | | **Female** | | .235 | .070 |

Note. Pearson product-moment correlation coefficients between total sample exploration and novelty preference index (discrimination ratio or oddity preference) are not significant unless

otherwise reported. In our experiments, the amount of object exploration does not consistently correlate with task performance.

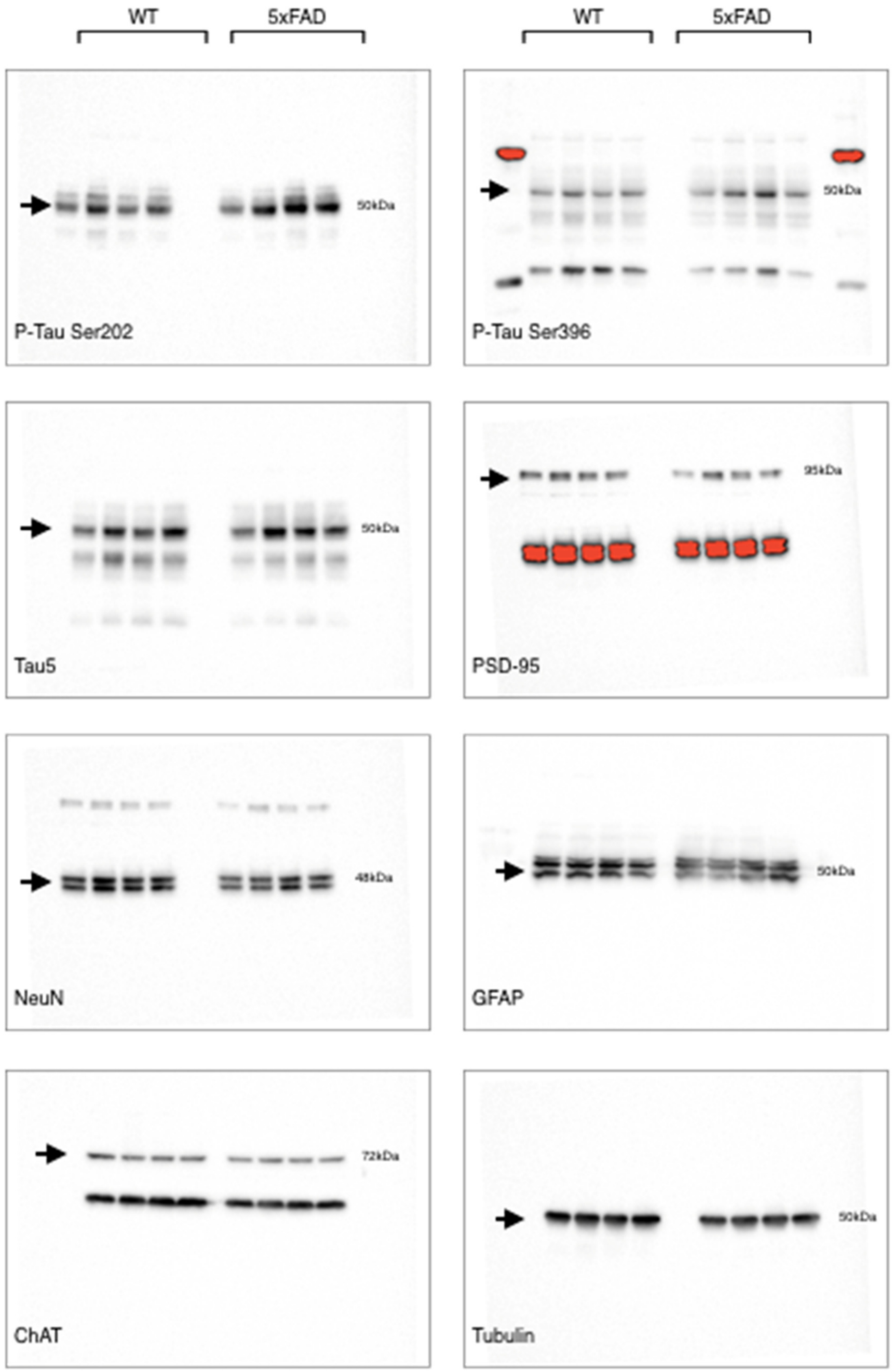


Supplementary Figure 1. Full-length representative western blots for 5xFAD male cortex. ChAT is shown with Tubulin. Each blot was probed for tubulin; here we show a representative sample. Arrows indicate the quantified band. No over-exposed bands were quantified.

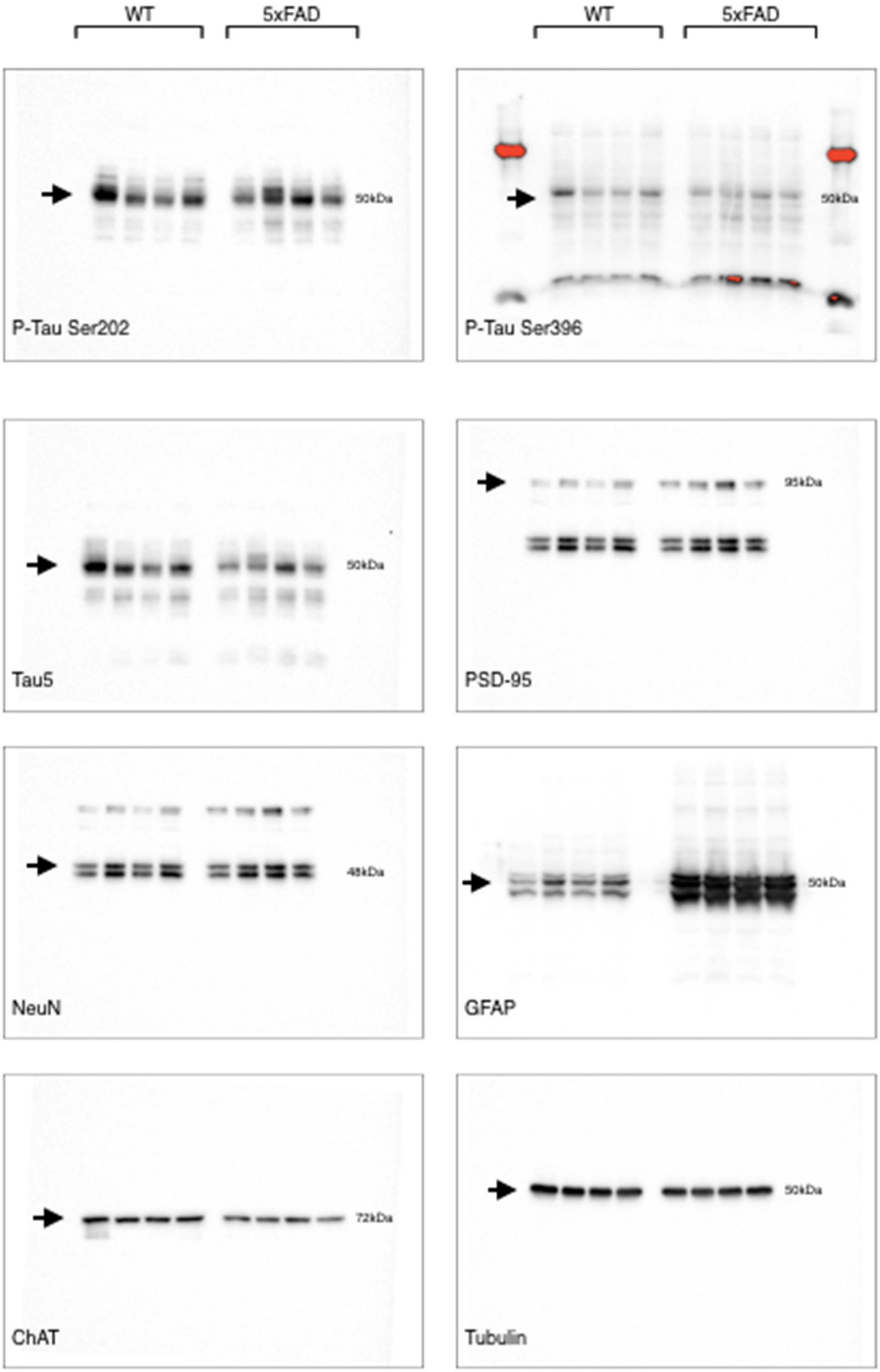


Supplementary Figure 2. Full-length representative western blots for 5xFAD female cortex. PSD-95 and NeuN were run on the same blot. Each blot was probed for tubulin; here we show a representative sample. Arrows indicate the quantified band. No over-exposed bands were quantified.

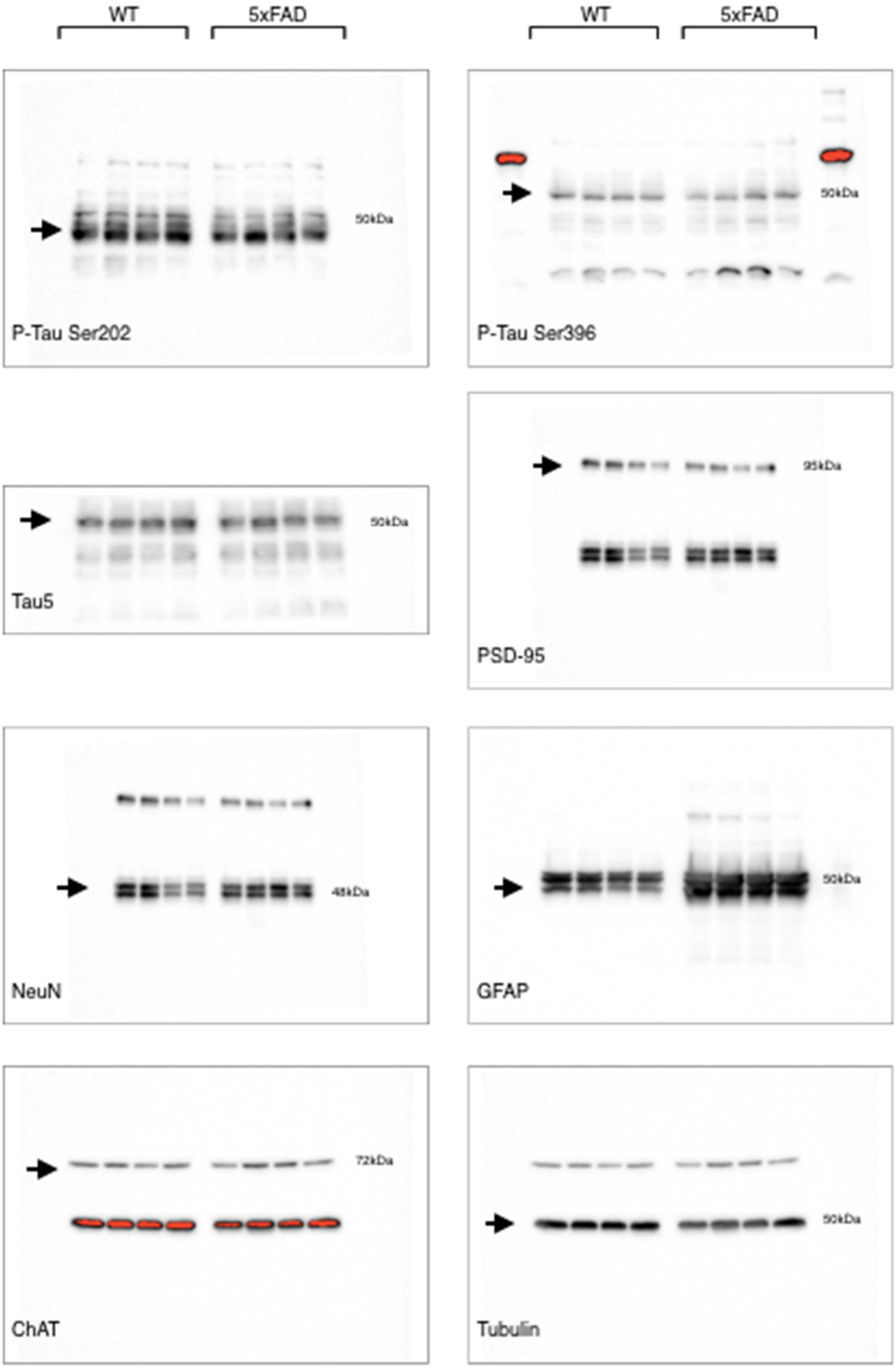


Supplementary Figure 3. Full-length representative western blots for 5xFAD male hippocampus. PSD-95 and NeuN were run on the same blot. ChAT is shown with tubulin with different exposure times. Each blot was probed for tubulin; here we show a representative sample. Arrows indicate the quantified band. No over-exposed bands were quantified.

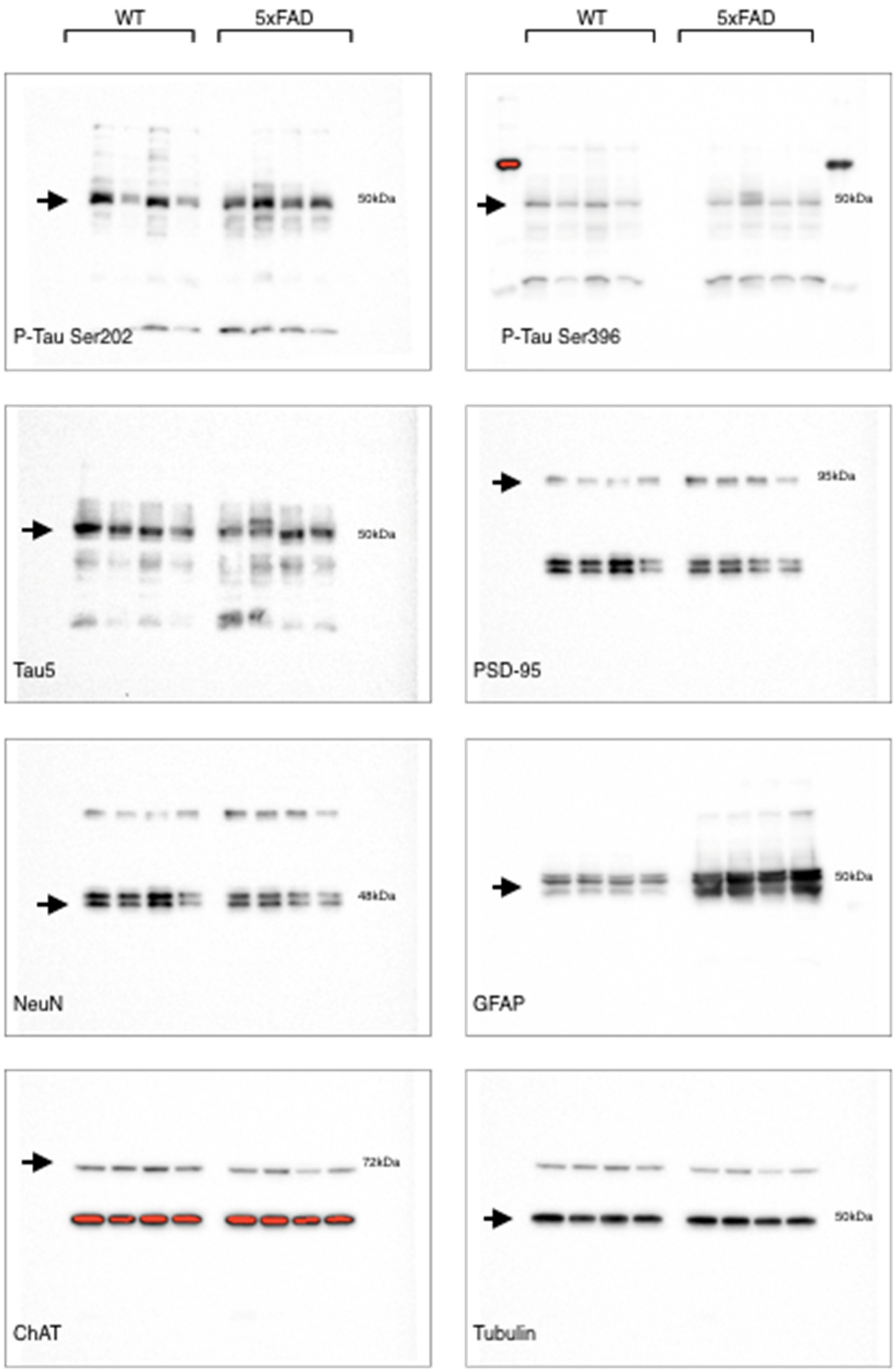


Supplementary Figure 4. Full-length representative western blots for 5xFAD female hippocampus. PSD-95 and NeuN were run on the same blot. ChAT is shown with tubulin with different exposure times. Each blot was probed for tubulin; here we show a representative sample. Arrows indicate the quantified band. No over-exposed bands were quantified.

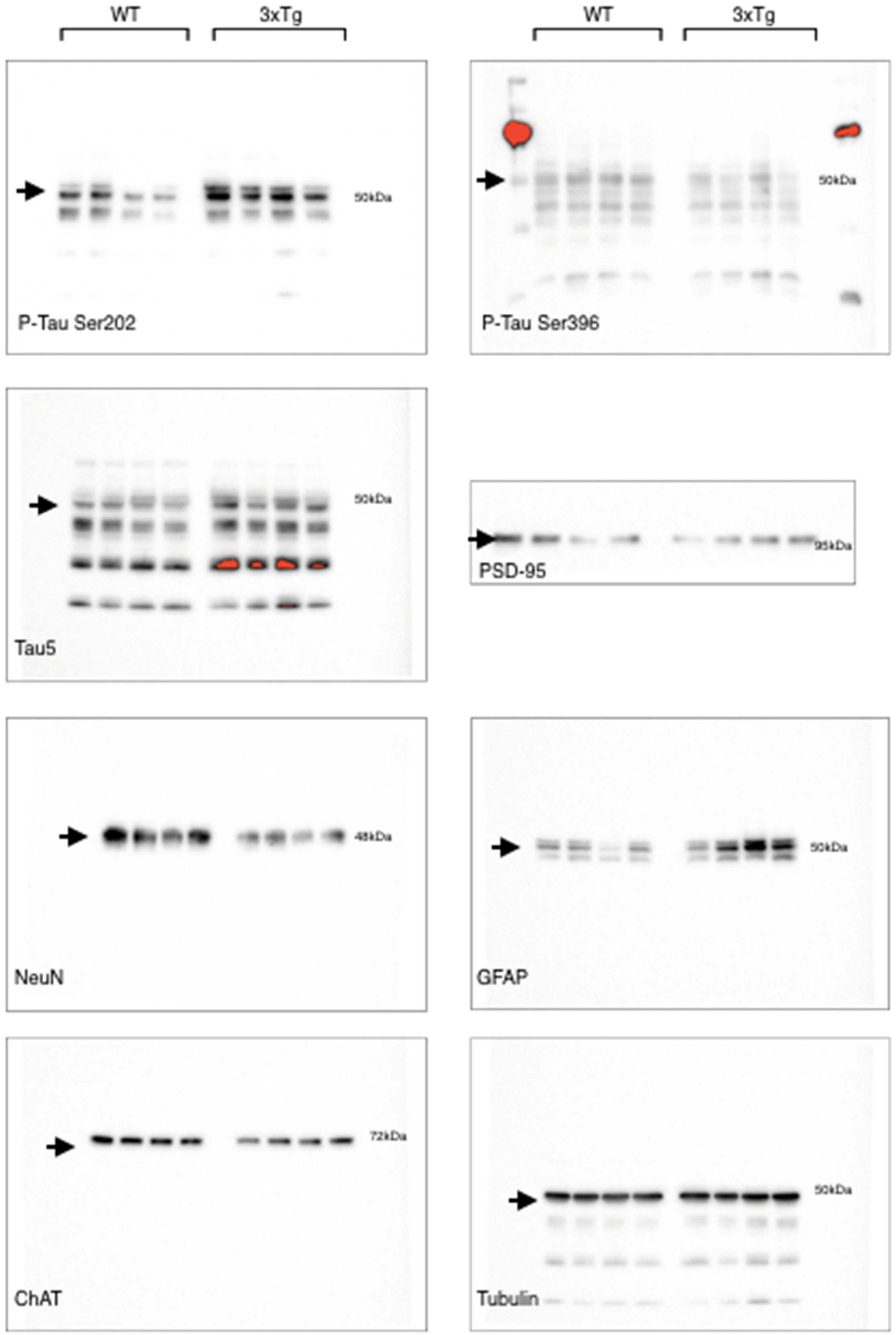


Supplementary Figure 5. Full-length representative western blots for 3xTG male cortex. Each blot was probed for tubulin; here we show a representative sample. Arrows indicate the quantified band. No over-exposed bands were quantified.

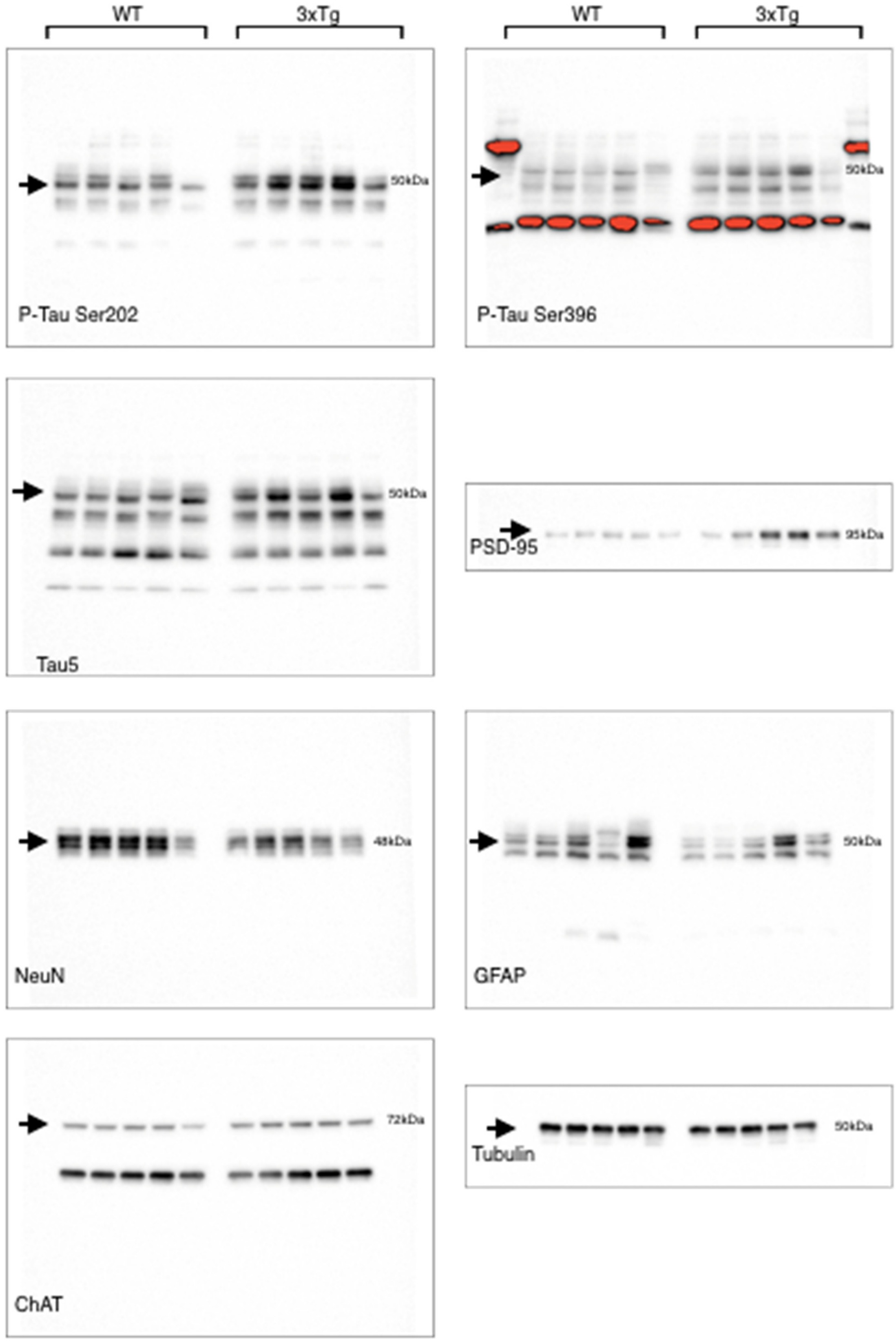


Supplementary Figure 6. Full-length representative western blots for 3xTG female cortex. ChAT is shown with tubulin. Each blot was probed for tubulin; here we show a representative sample. Arrows indicate the quantified band. No over-exposed bands were quantified.

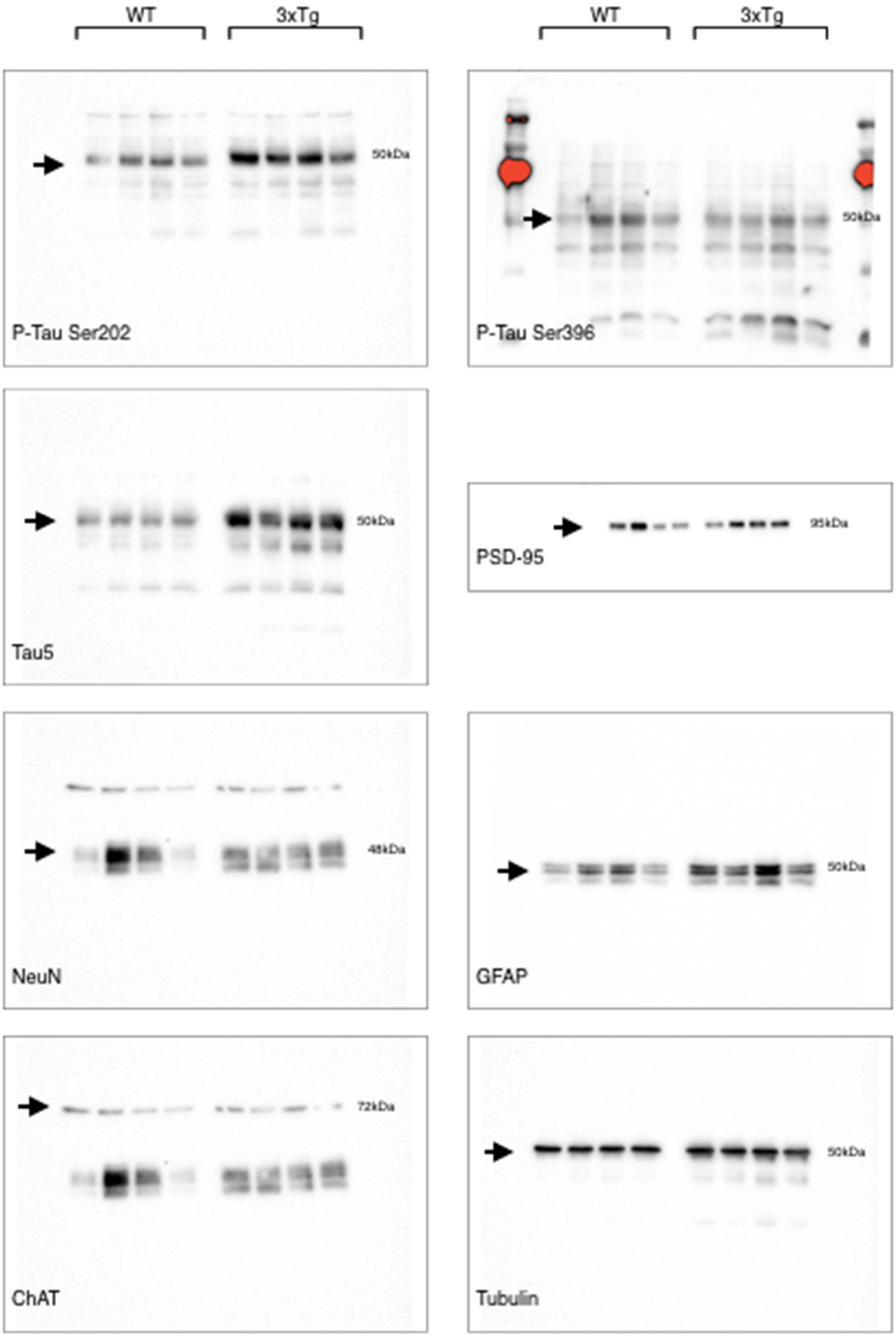

Supplementary Figure 7. Full-length representative western blots for 3xTG male hippocampus. NeuN and ChAT were run on the same blot. Each blot was probed for tubulin; here we show a representative sample. Arrows indicate the quantified band. No over-exposed bands were quantified.

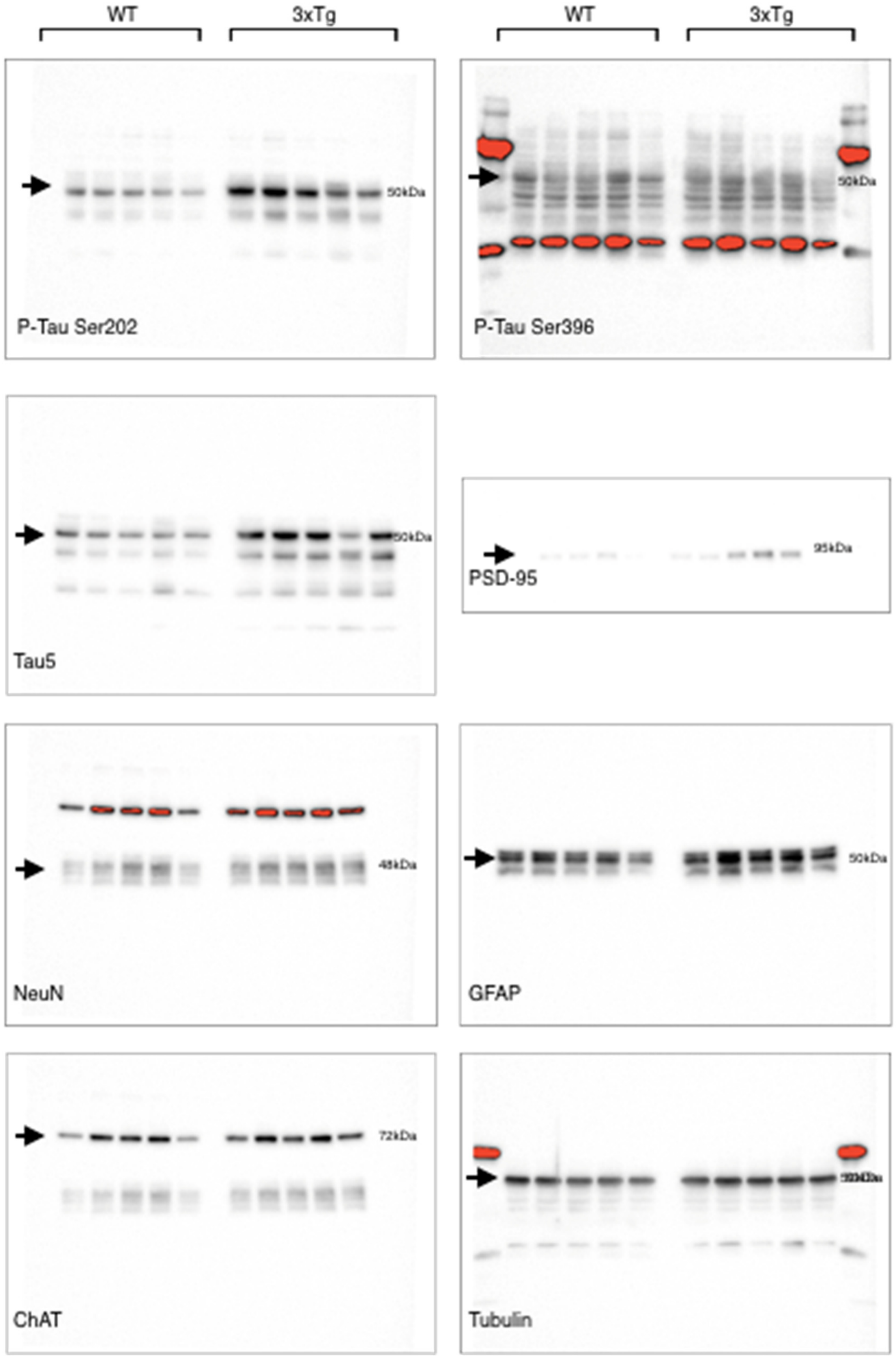


Supplementary Figure 8. Full-length representative western blots for 3xTG female hippocampus. NeuN and ChAT were run on the same blot with different exposure times. Each blot was probed for tubulin; here we show a representative sample. Arrows indicate the quantified band. No over-exposed bands were quantified.
